# Comparing prevalence and types of potentially inappropriate medications among patient groups in a post-acute and secondary care hospital

**DOI:** 10.1038/s41598-023-41617-0

**Published:** 2023-09-15

**Authors:** Hirotaka Nakashima, Hiromichi Ando, Hiroyuki Umegaki

**Affiliations:** 1 Department of Community Healthcare and Geriatrics, Nagoya University Graduate School of Medicine, 65 Tsurumai-Cho, Showa-Ku, Nagoya, Aichi 466-8560 Japan; 2 Wako-Kai Yamada Hospital, Gifu, Gifu Japan

**Keywords:** Geriatrics, Drug therapy, Rehabilitation

## Abstract

Reducing potentially inappropriate medications (PIMs) is a challenge in post-acute care hospitals. Some PIMs may be associated with patient characteristics and it may be useful to focus on frequent PIMs. This study aimed to identify characteristic features of PIMs by grouping patients as in everyday clinical practice. A retrospective review of medical records was conducted for 541 patients aged 75 years or older in a Japanese post-acute and secondary care hospital. PIMs on admission were identified using the Screening Tool for Older Person’s Appropriate Prescriptions for Japanese. The patients were divided into four groups based on their primary disease and reason for hospitalization: post-acute orthopedics, post-acute neurological disorders, post-acute others, and subacute. Approximately 60.8% of the patients were taking PIMs, with no significant difference among the four patient groups in terms of prevalence of PIMs (p = 0.08). However, characteristic features of PIM types were observed in each patient group. Hypnotics and nonsteroidal anti-inflammatory drugs were common in the post-acute orthopedics group, multiple antithrombotic agents in the post-acute neurological disorders group, diuretics in the post-acute others group, and hypnotics and diuretics in the subacute group. Grouping patients in clinical practice revealed characteristic features of PIM types in each group.

## Introduction

Medication management in older patients with multimorbidity can often be challenging. As comorbidity increases, so does the number of prescription medications, leading to a higher risk of using potentially inappropriate medications (PIMs)^1^. A previous study has reported that the prevalence of PIM use among hospitalized patients ranges from 30.4 to 97.1%^2^. Patients who are prescribed PIMs face an elevated risk of experiencing falls, adverse drug reactions, hospitalization, and even mortality^3^. This risk is particularly pronounced among older patients due to factors such as age-related physiological changes, frailty, and cognitive impairment^1^.

There are many reports on reducing PIMs^4–6^, and deprescribing protocols or algorithms are now available^7, 8^. Essentially, these strategies consist of a full medication review for each patient, and repeated personalized adjustment and assessment of medications. However, these methods often cannot be effectively implemented in all inpatients in a non-urban post-acute or secondary care hospital because of numerous barriers, including limited resources^9^. Indeed, the likelihood of being prescribed PIMs may increase during hospitalization^10^. A less burdensome and feasible approach is needed.

In Japan, most post-acute hospitals have various wards in addition to rehabilitation units, including post-acute transitional care wards and subacute wards. If differences in the prevalence and types of PIMs exist among these wards, it would provide insights into potential strategies for addressing PIMs that are better suited to each ward, consequently enhancing the hospital practices.

Previous studies have reported that the frequency of PIMs varies according to patient characteristics including comorbidities and the number of concurrent medications^11, 12^. The drug types of PIM may also vary based on patient characteristics. For example, a previous study has reported that hypnotics and antidepressants were common among patients undergoing hip fractures repair^13^. Similarly, patients transferred to rehabilitation hospitals after stroke frequently receive antipsychotics, hypnotics, and proton pump inhibitors^14^. However, most studies to date have focused on the types of PIMs within specific populations, with few studies exploring differences among patient groups.

Therefore, the aim of this study was to determine the characteristic features of PIMs prevalence and types according to patient group when inpatients were divided as in everyday clinical practice in a post-acute and secondary care hospital.

## Methods

### Study design

This retrospective cross-sectional study was based on a review of the medical records at Yamada Hospital, which is a 113-bed facility located in a suburban area in Gifu Prefecture, Japan. Almost half of the beds are for rehabilitation and the other half are for secondary-level acute or transitional care. Patients are transferred to our hospital from tertiary care centers for rehabilitation after acute inpatient treatment for conditions such as stroke and hip fracture, or they are admitted directly from the patient’s home or nursing home because of an acute disease such as pneumonia.

The study was approved by the Yamada Hospital ethics committee and was conducted in accordance with the principles of the Declaration of Helsinki. The need for informed consent for this study was waived by the Yamada Hospital ethics committee. This was because only data from medical records were used. However, the participants could withdraw from the study via the opt-out method by accessing the Yamada Hospital website. These methods were in accordance with the national guideline^15^. The recommendations of the Strengthening the Reporting of Observational Studies in Epidemiology (STROBE) statement were followed^16^.

### Participants

This study involved patients aged 75 years or older admitted to Yamada Hospital from January 1, 2021 to December 31, 2021. We focused on this age group because the PIMs criteria used in this study were for patients aged 75 years or older^17^. Patients who were still hospitalized on January 1, 2022 were excluded. For patients who had 2 or more admissions during the study period, only the first admission was included.

### Data collection

The first author collected the patient information from the electronic medical records, which included patient referral documents and nursing summaries from the referring hospital. Within our hospital, physicians follow a practice of making a list of problems (diseases) in the admission summary. The following data were collected: age, sex, place of residence before admission (home or nursing home), primary diagnosis, comorbidities, Charlson Comorbidity Index (CCI)^18^, height, weight, medications on admission, and daily functional status on admission. For medications on admission, we referred to medication identification records created by pharmacists in Yamada hospital. These records are routinely generated as part of their daily clinical practice. Daily functional status was assessed using the Functional Independence Measure^19^ and the Independence Scale of the Disabled Elderly (ISDE)^20^. According to the ISDE, patients are categorized into the following four groups based on a nurse’s clinical judgment: Rank J (independent), Rank A (house-bound), Rank B (chair-bound), and Rank C (bed-bound)^20^.

### Evaluation of medication

The total number of medications was counted for each patient. We included medications that were considered to be for transient use. We also included inhalants and patch medications for the treatment of internal diseases, but we excluded eye drops, nose drops, patch medications, and ointments for eye diseases, otolaryngological diseases, orthopedic diseases, and skin diseases. Intravenous or pro re nata medications were also excluded. We defined taking 5 or more medications as polypharmacy^21^. We evaluated PIMs according to the Screening Tool for Older Person’s Appropriate Prescriptions for Japanese (STOPP-J)^17^. Although other tools, such as the Beers criteria^22^, are available for evaluation of PIMs, we used STOPP-J because a previous study suggested that country-oriented criteria would be clinically useful^23^. According to STOPP-J, loop diuretics or aldosterone antagonists are deemed to be PIMs regardless of the patient’s condition. Thiazides were not considered to be PIMs.

### Classification of patients into groups

We divided patients into four groups based on the patient’s primary disease and reason for hospitalization. The four groups are subacute, post-acute orthopedics, post-acute neurological disorders, and post-acute others. Patients in the subacute group are directly admitted to our hospital from the patient’s home or nursing home for treatment of acute disease such as pneumonia. “Post-acute” in the present study means transfer from an acute hospital for rehabilitation or transitional care after acute inpatient treatment. In everyday practice at our hospital, a patient’s ward and treatment team are determined in this way.

In Japan, indications for hospitalization in convalescent rehabilitation wards are defined by the Ministry of Health, Labour and Welfare^24, 25^. Briefly, the indications contain three disease categories: neurological disorders, including stroke and spinal cord injury; orthopedic diseases, including hip fracture, pelvic fracture, and vertebral fracture; and disuse syndrome after surgery or pneumonia. In practice, patients are rarely transferred to rehabilitation wards for disuse syndrome, and more than 90% of rehabilitation wards in Japan are occupied mostly (> 80%) by patients needing rehabilitation for neurological disorders and orthopedic diseases^26^. In addition, approximately 30% of rehabilitation wards are occupied mostly (> 80%) by either neurological or orthopedic patients^26^. Approximately 90% of post-acute hospitals have wards in addition to rehabilitation wards, and patients who need rehabilitation or transitional care but do not meet the indications are admitted to these wards^27^. Therefore, the method used in this study of dividing patients into four groups is relatively standard in Japan.

### Outcome

The aim of the present study was to assess the differences in prevalence and types of PIMs among the patient groups. Thus, the main outcomes were the prevalence of PIMs in each of the four patient groups. The prevalence of PIMs was calculated for the entire category of PIMs, as well as for specific drug categories.

### Statistical analysis

The descriptive statistics were used to summarize the patient data.

We compared the four groups in terms of total number of medications, frequency of polypharmacy, total number of PIMs, and frequency of taking PIMs. We also compared the groups in terms of frequency of PIMs by medication category, limited to frequently taken medication categories (5% or more of all patients). Diuretics (loop diuretics and/or aldosterone antagonists) are sometimes appropriate for patients with heart failure. Therefore, we also analyzed the data for patients without heart failure who were taking diuretics. Furthermore, we investigated the frequency of proton-pomp inhibitors (PPIs) use. This was because PPIs are prescribed quite extensively in Japan, and while they are listed as PIMs according to the 2019 Beers criteria^22^, they are not included in STOPP-J^23^. It should be noted that PPIs were not included in the overall PIMs frequency calculation in the present study. Prior research has indicated a higher prescription rate of PIMs for female patients compared to male patients^11^. Therefore, we performed additional analyses to explore differences in the frequency of PIMs based on sex, both within the entire patient cohort and within each of the four patient groups. These comparisons were conducted using one-way analysis of variance, the Kruskal–Wallis test, or Fisher’s exact test as appropriate.

Furthermore, to investigate the patient characteristics associated with the use of PIMs, both crude and adjusted logistic regression analyses were performed. The adjusted model included, with reference to previous study^11^, age, sex, living situation before hospitalization (at home or elsewhere), daily functional status (ISDE category), total number of medications, CCI, and the four patient groups. We also conducted similar logistic analyses for benzodiazepine receptor agonists (BZRAs, including benzodiazepines and so-called Z-drugs), because they are one of the most common and important PIMs.

All statistical analyses were performed using EZR version 1.55 (Saitama Medical Center, Jichi Medical University, Saitama, Japan). EZR is a graphical user interface for R version 4.1.2 (The R Foundation for Statistical Computing, Vienna, Austria)^28^. A sample size calculation was not conducted a priori for this study. This decision was made based on our belief that any findings detectable in data from one year would hold significance in clinical practice on a ward-by-ward basis or within small hospitals. The analyses were performed without imputation of missing values. A p-value of less than 0.05 was considered statistically significant.

## Results

**Figure 1:**
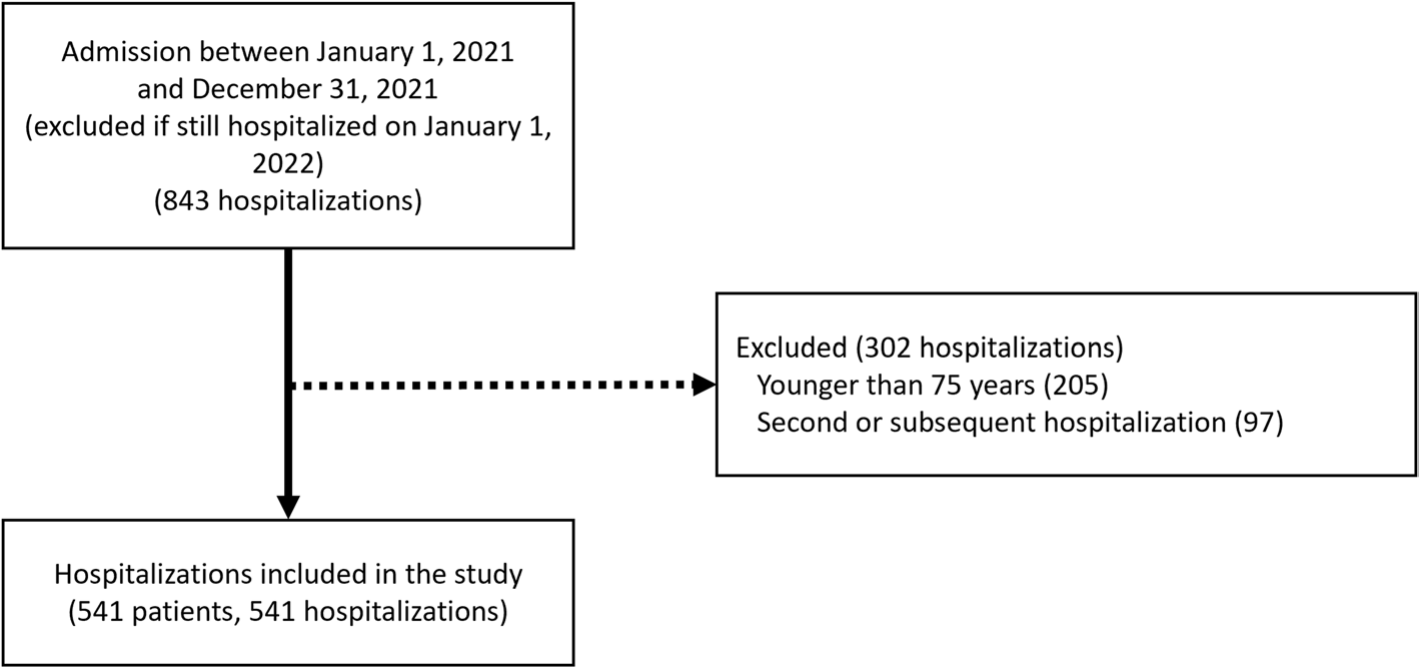
Flowchart of patient selection.

**Figure 2:**
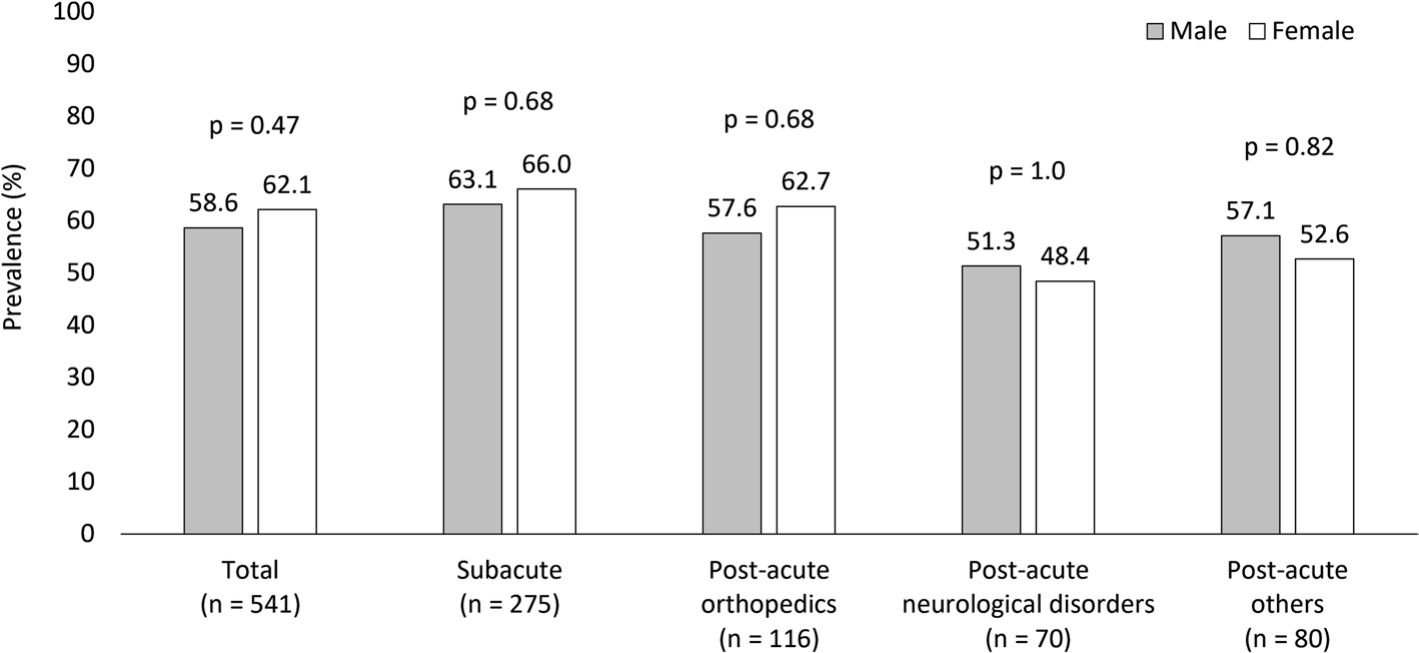
Differences in the frequency of PIMs use based on sex.

In total, 541 patients were included in the study (Fig. 1). Median age was 86 (81–90) years (Table 1). Most patients were female (63.4%), and most lived in their home before hospitalization (62.7%) but were chair-bound or bedridden on admission to Yamada Hospital. The patients were taking a median of 7 medications on admission; 74.5% were on polypharmacy. About 60.8% of patients were taking PIMs, and there was no difference by sex in the frequency of PIMs use (p = 0.47) (Fig. 2). The most frequent PIMs were diuretics (loop diuretics and/or aldosterone antagonists; 25.1%), BZRAs (17.7%), nonsteroidal anti-inflammatory drugs (NSAIDs; 10.3%), oral antidiabetic agents (9.4%), antipsychotics (7.8%), and antithrombotic agents (6.5%) (Table 1).

**Table 1:** Demographic and clinical characteristics and medications according to patient group.

	Total		Subgroup								p^a^
			Subacute		Post-acute orthopedics		Post-acute neurological disorders		Post-acute others		
Patients	541	(100)	275	(50.8)	116	(21.4)	70	(12.9)	80	(14.8)	–
Age, years (n = 541)	86	(81–90)	87	(83–92)	86	(81–90)	82	(78–88)	85	(81–87)	< 0.001
Male sex (n = 541)	198	(36.6)	84	(30.5)	33	(28.4)	39	(55.7)	42	(52.5)	< 0.001
Living at home before hospitalization (n = 541)	339	(62.7)	102	(37.1)	105	(90.5)	66	(94.3)	66	(82.5)	< 0.001
Body mass index, kg/m^2^ (n = 531^b^)	20.0	3.6	19.6	3.8	19.9	2.9	21.9	3.5	19.6	3.3	< 0.001
ISDE^c^ (n = 538)	B	(B–C)	B	(B–C)	B	(B–C)	B	(B–B)	B	(B–C)	< 0.001
FIM^d^ on admission (n = 483^e^)	46	(27–70)	38	(22–62)	68	(44–82)	49	(28–75)	42	(26–60)	< 0.001
Length of hospital stay, days (n = 541)	36	(21–51)	25	(14–41)	48	(36–59)	60	(38–83)	39	(24–49)	< 0.001
Charlson comorbidity Index^f^ (n = 541)	2	(1–3)	2	(1–3)	1	(0–1)	3	(2–4)	2	(1–4)	< 0.001
Comorbidities (n = 541)											
Hypertension	325	(60.1)	156	(56.7)	68	(58.6)	56	(80)	45	(56.3)	0.003
History of myocardial infarction	40	(7.4)	17	(6.2)	11	(9.5)	4	(5.7)	8	(10)	0.48
Heart failure	133	(24.6)	86	(31.3)	9	(7.8)	9	(12.9)	29	(36.3)	< 0.001
Atrial fibrillation	78	(14.4)	44	(16.0)	9	(12.9)	9	(7.8)	16	(20.0)	0.06
Peripheral arterial disease or aortic aneurysm	19	(3.5)	8	(2.9)	1	(0.9)	4	(5.7)	6	(7.5)	0.047
Stroke or paralysis	184	(34.0)	74	(26.9)	18	(15.5)	67	(95.7)	25	(31.3)	< 0.001
Dementia	217	(40.1)	150	(54.5)	11	(9.5)	26	(37.1)	30	(37.5)	< 0.001
Chronic pulmonary diseases	37	(6.8)	14	(5.1)	10	(8.6)	4	(5.7)	9	(11.3)	0.21
Rheumatic diseases	21	(3.9)	9	(3.3)	6	(5.2)	2	(2.9)	4	(5.0)	0.71
Peptic ulcer	12	(2.2)	6	(2.2)	2	(1.7)	2	(2.9)	2	(2.5)	0.92
Chronic hepatitis or cirrhosis	14	(2.6)	5	(1.8)	1	(0.9)	2	(2.9)	6	(7.5)	0.032
Diabetes mellitus	132	(24.4)	66	(24.0)	27	(23.3)	20	(28.6)	19	(23.8)	0.85
Renal dysfunction (Cre > 3.0 mg/dL)	9	(1.7)	9	(3.3)	0	(0)	0	(0)	0	(0)	0.052
Solid tumor	15	(2.8)	20	(7.3)	3	(2.6)	2	(2.9)	16	(20.0)	< 0.001
Leukemia or lymphoma	6	(1.1)	4	(1.5)	1	(0.9)	0	(0)	1	(1.3)	0.93
Acquired immune deficiency syndrome	0	(0)	0	(0)	0	(0)	0	(0)	0	(0)	-
Total number of medications (n = 541)	7	(4–9)	7	(5–9)	6	(4–9)	6	(4–9)	7	(5–11)	0.18
Polypharmacy^g^ (n = 541)	403	(74.5)	211	(76.7)	85	(73.3)	47	(67.1)	60	(75.0)	0.41
Total number of PIMs (n = 541)	1	(0–2)	1	(0–2)	1	(0–2)	0.5	(0–2)	1	(0–2)	0.07
Use of any PIMs (n = 541)	329	(60.8)	179	(65.1)	71	(61.2)	35	(50.0)	44	(55.0)	0.08
Antipsychotics	42	(7.8)	28	(10.2)	6	(5.2)	3	(4.3)	5	(6.3)	0.24
Benzodiazepines or Z-drugs	96	(17.7)	57	(20.7)	25	(21.6)	7	(10.0)	7	(8.8)	0.014
Antidepressants	0	(0)									
Sulpiride	3	(0.6)	3	(1.1)	0	(0.0)	0	(0.0)	0	(0.0)	
Antiparkinsonian drugs (anticholinergic)	1	(0.2)	1	(0.4)	0	(0.0)	0	(0.0)	0	(0.0)	
Oral steroids	0	(0)									
Antithrombotic drugs (multiple use of them)	35	(6.5)	8	(2.9)	3	(2.6)	16	(22.9)	8	(10.0)	< 0.001
Digitalis	0	(0)									
Loop diuretics and/or aldosterone antagonists^h^	136	(25.1)	89	(32.4)	14	(12.1)	8	(11.4)	25	(31.3)	< 0.001
Without heart failure	49	(9.1)	34	(12.4)	9	(7.8)	3	(4.3)	3	(3.8)	
Beta-blockers	0	(0)									
Alpha-1-blockers	21	(3.9)	13	(4.7)	3	(2.6)	3	(4.3)	2	(2.5)	
H1 receptor antagonists (first generation)	2	(0.4)	2	(0.7)	0	(0.0)	0	(0.0)	0	(0.0)	
H2 receptor antagonists	25	(4.6)	11	(4.0)	8	(6.9)	4	(5.7)	2	(2.5)	
Antiemetic drugs	3	(0.6)	2	(0.7)	1	(0.9)	0	(0.0)	0	(0.0)	
Laxative (magnesium oxide)	14	(2.6)	10	(3.6)	0	(0.0)	1	(1.4)	3	(3.8)	
Oral antidiabetic drugs	51	(9.4)	26	(9.5)	14	(12.1)	8	(11.4)	3	(3.8)	0.19
Insulin (sliding scale)	10	(1.9)	0	(0.0)	1	(0.9)	5	(7.1)	4	(5.0)	
Overactive bladder drugs (anticholinergic)	14	(2.6)	8	(2.9)	4	(3.4)	1	(1.4)	1	(1.3)	
Nonsteroidal anti-inflammatory drugs	57	(10.5)	26	(9.5)	24	(20.7)	1	(1.4)	6	(7.5)	< 0.001
Proton-pump inhibitors	194	(35.9)	94	(34.9)	29	(25.0)	44	(62.9)	25	(31.3)	< 0.001

Data are presented as number (%), mean ± standard deviation, or median (interquartile range) unless indicated otherwise.

*Cre* creatinine, *FIM* Functional Independence Measure, *ISDE* Independence Scale of the Disabled Elderly, *PIMs* potentially inappropriate medications.

^a^Comparison among the four subgroups.

^b^Data on both height and weight were missing in 7 patients, and data on only height were missing in 3 patients.

^c^In ISDE, all patients were categorized into four groups: Rank J (independent), Rank A (house-bound), Rank B (chair-bound), and Rank C (bed-bound).

^d^FIM ranges from 18 to 126; a higher score indicates better function.

^e^Data on FIM were missing in 58 of 541 patients overall, in 56 of 275 patients in the post-acute group, and 2 of 80 patients in the post-acute others group.

^f^Charlson Comorbidity Index ranges from 0 to 37; a higher score indicates more comorbidities.

^g^Polypharmacy was defined as 5 or more medications.

^h^Thiazides were not considered to be PIMs in this study.

When comparing the four patient groups, significant differences in patient characteristics were observed (Table 1). The subacute group had a higher proportion of females, were less likely to be living at home prior to hospitalization, and had a higher prevalence of heart failure and dementia. The post-acute orthopedics group had a higher proportion of females, greater physical function (FIM), and had fewer comorbidities. Patients in the post-acute neurological disorders group were younger, had a higher body mass index, and a higher CCI (almost all patients had stroke). The post-acute others group had a higher prevalence of heart failure and malignant tumors.

The medications taken in the four patient groups are compared in Table 1. There were no significant differences among the four patient groups in the total number of medications (p = 0.18) or prevalence of PIMs (p = 0.08). However, characteristic features of PIM types were observed in each patient group. Patients in the subacute group were taking BZRAs (20.7%) and diuretics (32.4%) more frequently than patients in the other groups. The post-acute orthopedics group was frequently taking BZRAs (21.6%) and NSAIDs (20.7%), the post-acute neurological disorders group was taking 2 or more antithrombotic agents (22.9%), and the post-acute others group were taking diuretics (31.3%). In additional analyses on diuretics, the prevalence of diuretic use without heart failure was highest (12.4%) in the subacute group. The frequency of taking PPIs was higher overall (35.9%), particularly in the post-acute neurological disorders group (62.9%). There was no sex difference in the frequency of PIMs use within each patient group (Fig. 2). Table S1 of the Supplementary Information shows the most common primary diseases in the four patient groups. The most common medications and PIMs are shown in Supplementary Tables S2 and S3, respectively. The details of multiple use of antithrombotic agents are described in Table 2.

**Table 2:** Details of multiple use of antithrombotic agents.

	Total (n = 541)	Subgroup			
		Subacute (n = 275)	Post-acute orthopedics (n = 116)	Post-acute neurological disorders (n = 70)	Post-acute others (n = 80)
Dual antiplatelet therapy, n	21	2	2	12	5
One antiplatelet and one anticoagulant, n	12	6^a^	1^b^	2^c^	3^d^
Three or more antithrombotic agents, n	2	0	0	2^e^	0
Total, n	35	8	3	16	8
Anticoagulant use, n	13	6	1	3	3
With atrial fibrillation, n	7	3	0	2	2
With other reasons, n	2	1f.	0	0	1^ g^
Without clear indication, n	4	2	1	1	0

^a^All anticoagulants were direct oral anticoagulants (DOAC).

^b^Antiplatelet plus warfarin.

^c^Regarding anticoagulants, one patient was taking DOAC, and the other patient was taking warfarin.

^d^Regarding anticoagulants, two patients were taking DOAC, and one patient was taking warfarin.

^e^One patient was taking aspirin, clopidogrel, and cilostazol, and the other patient was taking these three antiplatelets and DOAC.

^f^DOAC for deep vein thrombosis.

^g^Warfarin for mechanical aortic valve.

Logistic regression analyses revealed that only the total number of medications was associated with the use of PIMs on admission (adjusted odds ratio (AOR) 1.51, 95% confidence interval (CI) 1.40–1.64) (Table 3). With regard to the use of BZRAs, the total number of medications (AOR 1.30, 95%CI 1.20–1.40) and patient group (subacute group, AOR 2.98, 95%CI 1.14–7.80; post-acute orthopedics group, AOR 3.95, 95% CI 1.43–10.90; the reference being the post-acute others group) showed significant associations (Table 4).

**Table 3:** Factors associated with the use of PIMs on admission (n = 538^a^).

Patient characteristics	Use of any PIMs, n (%)	Crude odds ratio (95% CI)	Adjusted odds ratio (95% CI)^d^
Age, per 1-year increase	–	1.00 (0.97, 1.03)	1.01 (0.98, 1.05)
Sex			
Female	213 (62.1)	Reference	Reference
Male	116 (58.6)	0.86 (0.60, 1.23)	0.85 (0.53, 1.34)
Living situation before hospitalization			
Home	198 (58.4)	Reference	Reference
Other	131 (64.9)	1.31 (0.92, 1.88)	1.37 (0.79, 2.39)
ISDE^b^, per 1-rank worse	–	0.77 (0.59, 0.99)*	0.74 (0.53, 1.03)
Total number of medications, per one more medication	–	1.50 (1.39, 1.61)***	1.51 (1.40, 1.64)***
CCI^c^, per 1-point higher	–	1.15 (1.04, 1.27)**	1.08 (0.96, 1.23)
Patient group			
Post-acute neurological disorders	35 (50.0)	Reference^e^	Reference^e^
Post-acute orthopedics	71 (61.2)	1.58 (0.87, 2.87)	1.56 (0.74, 3.29)
Post-acute others	44 (55.0)	1.22 (0.64, 2.33)	0.84 (0.38, 1.85)
Subacute	179 (65.1)	1.86 (1.10, 3.17)*	1.52 (0.75, 3.09)

*CCI* Charlson Comorbidity Index, *CI* confidence interval, *ISDE* Independence Scale of the Disabled Elderly, *PIMs* potentially inappropriate medications.

*^,^**^,^***Represent p < 0.05, p < 0.01, and p < 0.001, respectively.

^a^Out of a total of 541 patients, 3 patients with no ISDE data were excluded.

^b^In ISDE, all patients were categorized into four groups: Rank J (independent), Rank A (house-bound), Rank B (chair-bound), and Rank C (bed-bound).

^c^CCI ranges from 0 to 37; a higher score indicates more comorbidities.

^d^Adjusted model includes age, sex, living situation before hospitalization, ISDE, total number of medications, CCI, and patient group.

^e^Post-acute neurological disorders group was selected as a reference because this group had the lowest frequency of PIMs.

**Table 4:** Factors associated with the use of benzodiazepine receptor agonists (BZRAs)^a^ on admission (n = 538^b^).

Patient characteristics	Use of BZRAs, n (%)	Crude odds ratio (95% CI)	Adjusted odds ratio (95% CI)^e^
Age, per 1-year increase	–	1.01 (0.97, 1.05)	1.01 (0.97, 1.05)
Sex			
Female	70 (20.4)	Reference	Reference
Male	26 (13.1)	0.59 (0.36, 0.96)*	0.66 (0.38, 1.14)
Living situation before hospitalization			
Home	55 (16.2)	Reference	Reference
Other	41 (20.3)	1.31 (0.84, 2.06)	1.50 (0.81, 2.79)
ISDE^c^, per 1-rank worse	–	0.67 (0.49, 0.91)*	0.70 (0.49, 1.01)
Total number of medications, per one more medication	–	1.25 (1.17, 1.34)***	1.30 (1.20, 1.40)***
CCI^d^, per 1-point higher	–	0.99 (0.88, 1.11)	0.95 (0.82, 1.10)
Patient group			
Post-acute others	7 (8.8)	Reference^f^	Reference^f^
Post-acute orthopedics	25 (21.6)	2.86 (1.17, 7.00)*	3.95 (1.43, 10.90)**
Post-acute neurological disorders	7 (10.0)	1.16 (0.39, 3.48)	1.94 (0.59, 6.38)
Subacute	57 (20.7)	2.73 (1.19, 6.24)*	2.98 (1.14, 7.80)*

*CCI* Charlson Comorbidity Index, *CI* confidence interval, *ISDE* Independence Scale of the Disabled Elderly.

*^,^**^,^***Represent p < 0.05, p < 0.01, and p < 0.001, respectively.

^a^BZRAs include benzodiazepines and Z-drugs.

^b^Out of a total of 541 patients, 3 patients with no ISDE data were excluded.

^c^In ISDE, all patients were categorized into four groups: Rank J (independent), Rank A (house-bound), Rank B (chair-bound), and Rank C (bed-bound).

^d^CCI ranges from 0 to 37; a higher score indicates more comorbidities.

^e^Adjusted model includes age, sex, living situation before hospitalization, ISDE, total number of medications, CCI, and patient group.

^f^Post-acute others group was selected as a reference because this group had the lowest frequency of BZRAs use.

## Discussion

This study found a high prevalence of polypharmacy and PIMs in older inpatients admitted to a post-acute and secondary care hospital. When the patients were divided into four groups based on their primary disease and reason for hospitalization in the same way as in everyday clinical practice, there was no difference in the total number of medications or in the prevalence of PIMs among the groups. However, types of PIMs showed characteristic features in each group.

### PIMs in all patients

In this study, 60.8% of patients met the STOPP-J criteria for PIMs. This figure is within the range of 42.3%–72.4% reported in previous studies that have used STOPP-J^29–32^. The PIMs most frequently used in our study were also in accordance with those studies^29–32^.

There were no sex differences in the use of PIMs on admission in the present study. However, it is plausible that statistically significant differences could emerge with an increase in the number of participants. Previous studies reporting sex differences in the frequency of PIMs were conducted on a large scale (n > 250,000)^33, 34^.

As the determinant of the use of PIMs on admission, solely the total number of medications was extracted. A prior systematic review has also identified the number of medications as the most prevalent factor associated with PIMs use^11^, and the result of the present study is concurrent with that finding.

Regarding the differences in frequency and types of PIM in post-acute or secondary care hospitals when patients are categorized into multiple groups, we were unable to identify any comparable studies to benchmark against the present study. If the number of participants were slightly larger, there might be differences in the frequency of PIMs among the patient groups.

### Subacute group

Patients in this group were frequently taking diuretics (loop diuretics and/or aldosterone antagonists) (32.4%). Diuretics can cause several complications, including falls, fractures, dehydration, and electrolyte imbalance^17^. In previous studies that used the STOPP-J criteria, 12.1%–25.6% of patients were taking diuretics^23, 29–31^. The difference in diuretics use in our study may reflect the prevalence of heart failure. However, it should be noted that 12.4% of patients in our subacute group were taking diuretics without a diagnosis of heart failure in contrast to 3.8% in the post-acute others group. This suggests that many patients in the subacute group were taking diuretics without a clear indication, or were not recognized as having heart failure^35^. Special attention may be necessary when an unexpectedly hospitalized older patient is taking diuretics.

### Post-acute orthopedics group

Many patients in this group were taking BZRAs (21.6%) on admission. The prevalence of BZRA use in this group was almost the same as that in the subacute group (20.7%) but was higher than that in the other two groups (approximately 9 to 10%) [It should be noted that this difference may not be truly statistically significant due to multiple comparisons and a relatively high p-value (0.014)]. A previous study has reported that females have higher odds of being prescribed benzodiazepines^36^. Interestingly, a higher proportion of females was noted in both the post-acute orthopedics group and the subacute group. However, the results from the multiple logistic regression analyses in the present study indicated that the use of BZRAs was more closely associated with patient group rather than sex. Another previous study reported that no medication adjustments were made during hospital stays on a conventional trauma ward^37^. BZRAs may have been discontinued before referral to our hospital in the patients in post-acute neurological disorders and post-acute others groups, which might explain their lower BZRA use. Many patients in the post-acute orthopedics group were hospitalized because of fall-related fractures. As is well known, BZRAs are associated with adverse events such as falls^38^. Discontinuation of BZRAs is especially important in this population.

### Post-acute neurological disorders group

Patients in this group were frequently taking multiple antithrombotic medications (22.9%). Most patients (13 out of 16 patients) were taking concomitant antiplatelet medications without any anticoagulants (Table 2). At least in the acute phase, the benefits would have outweighed the risks. However, it is better to consider reducing those medications during or after hospitalization, depending on the patient’s condition, because of the potential risk of bleeding^39, 40^. We collected data on the details of the antithrombotic medications and on the diagnostic names. Unfortunately, however, we did not gathered data regarding the length of stay at the referring hospital or the number of days since stroke onset. Consequently, we were unable to thoroughly assess the appropriateness of the multiple antithrombotic medications at the time of admission to our hospital. Rather, considering the frequency of prescriptions and clinical importance, the findings of the current study might indicate a necessity for intervention against hypnotics and PPIs.

### Post-acute others group

Many patients in this group were taking diuretics (31.3%), and most (22 out of 25 who were taking diuretics) had heart failure. This finding suggests that, unlike in the subacute group, most patients in the post-acute others group were prescribed diuretics based on clinical necessity, and these patients were identified as having heart failure. However, diuretics should be used at the smallest dose possible with monitoring for dehydration and electrolyte abnormalities^41^.

### Implications for clinical practice and healthcare policy

The findings of this study might not be revolutionary; however, they do hold implications for clinical practice. Hospitals sharing similar characteristics with ours could focus on the PIMs identified in the present study. Other hospitals could develop more practical PIMs countermeasures by grouping patients in a manner suitable to each hospital and identifying the most common PIMs (which would not be overly burdensome in itself). This approach might be more feasible than intervening equally for all PIMs in hospitalized patients.

The present study also has implications for healthcare policy. Since 2016, Japan has been implementing a policy that offers incentives to hospitals that succeed in reducing two or more medications per patient^42^. As a result of this policy, the prevalence of polypharmacy seems to have decreased^42^. However, conversely, the prevalence of PIMs has not decreased; in fact, it has increased^43^. A recent review indicated that healthcare policies aimed at promoting the deprescribing of specific PIMs might lead to unintended consequences^44^. One potential approach could involve assisting in the investigation and intervention of PIMs on a hospital-by-hospital or ward-by-ward basis. This would also enhance the staff's sense of participation in combating against PIMs, compared to if the government were to take the lead in reducing specific PIMs.

### Limitations

This study has several limitations. If we had used other criteria for PIMs, such as the 2019 Beers criteria, the frequently taken PIMs might have been different from those identified in the present study. For instance, under the 2019 Beers criteria, multiple antithrombotic medications are not classified as PIMs. However, under the criteria, PPIs are considered as PIMs, and it would form the characteristic features of PIMs in each patient group. In essence, even with variations in PIMs criteria, the primary conclusion might remain unchanged, that is, each patient group would have characteristic features in types of PIMs. Another limitation is that this study was conducted at a single center. Therefore, caution is necessary when generalizing its results. Patients in another hospital may have to be divided in another way, and common PIMs might differ by hospital and country. Especially, providing subacute care, rehabilitation care, and other post-acute transitional care within a single hospital might be specific to Japan. However, the results from the present study suggest that grouping patients in a way suitable to each hospital may generally be helpful in understanding the status of PIMs.

## Conclusion

In this study, we found a high prevalence of PIMs in older inpatients on admission in a post-acute and secondary care hospital. When we divided patients into four groups based on actual clinical practice, there was no difference in the prevalence of PIMs among the groups, but types of PIMs showed characteristic features in each group.

### Supplementary Information

Supplementary Tables.

## Data Availability

The datasets generated during and/or analysed during the current study are not publicly available due to patient privacy considerations, but are available from the corresponding author on reasonable request.
