# Supplementary material for: Comparing prevalence and types of potentially inappropriate medications among patient groups in a post-acute and secondary care hospital
Source: Sci Rep. 2023 Sep 15;13:14543. doi: 10.1038/s41598-023-41617-0 (PMC10504332; doi:10.1038/s41598-023-41617-0)
Supplement: Supplementary file 1 — Supplementary Tables. [file 41598_2023_41617_MOESM1_ESM.pdf]

Supplementary Information

Article title

Comparing prevalence and types of potentially inappropriate medications among patient groups in a post-acute and secondary care hospital

Journal name

scientific reports

Author names

Hirotaka Nakashima, Hiromichi Ando, Hiroyuki Umegaki.

Corresponding author

Hirotaka Nakashima

Department of Community Healthcare and Geriatrics, Nagoya University Graduate School of Medicine, Nagoya, Aichi, Japan

naka621@med.nagoya-u.ac.jp

Table S1: Most common primary diseases in the four patient groups

Table S2: Most common medications according to patient group

Table S3: Most common potentially inappropriate medications according to patient group

Table S1 Most common primary diseases in the four patient groups

	Diagnosis	n	%
Subacute	Pneumonia	73	26.5
(n=275)	Vertebral compression fracture	23	8.4
	Heart failure	17	6.2
	Urinary tract infections	16	5.8
	Loss of appetite or dysphagia	15	5.5
	Orthopedic diseases other than fractures[a]	13	4.7
	Cancer and complications	8	2.9
	Disuse syndrome	8	2.9
	Dementia	6	2.2
	Diabetes mellitus and complications	6	2.2
Post-acute orthopedics	Hip fracture	73	62.9
(n=116)	Vertebral compression fracture	10	8.6
	Fractures other than hip, vertebrae, and pelvis	9	7.8
	Postoperative for spinal diseases other than vertebral compression fracture	7	6.0
	Pelvic fracture	5	4.3
Post-acute neurological disorders	Cerebral infarction	48	68.6
(n=70)	Cerebral hemorrhage	12	17.1
	Subarachnoid hemorrhage	6	8.6
	Postoperative for chronic subdural hematoma	1	1.4
	Postoperative for brain abcess	1	1.4
Post-acute others	Disuse syndrome	54	67.5
(n=80)	Heart failure	5	6.3
	Cancer and complications	5	6.3
	Non-infectious pneumonia	2	2.5
	Rheumatic diseases	2	2.5

[a]Includes spinal canal stenosis (n=4), osteoarthritis (n=3), calcium pyrophosphate dehydrate deposition disease (n=2), low back pain (n=2), joint dislocation (n=1), contusion (n=1)

Table S2 Most common medications according to patient group

Total (n=541)			Subgroup											
			Subacute (n=275)			Post-acute orthopedics (n=116)			Post-acute neurological disorders (n=70)			Post-acute others (n=80)		
Amlodipine	187	34.6%	Amlodipine	100	36.4%	Amlodipine	39	33.6%	Lansoprazole	39	55.7%	Magnesium oxide	33	41.3%
Lansoprazole	156	28.8%	Magnesium oxide	82	29.8%	Acetaminophen	38	32.8%	Amlodipine	30	42.9%	Lansoprazole	19	23.8%
Magnesium oxide	153	28.3%	Lansoprazole	81	29.5%	Magnesium oxide	26	22.4%	Aspirin	24	34.3%	Amlodipine	18	22.5%
Acetaminophen	83	15.3%	Bisoprolol	52	18.9%	Celecoxib	23	19.8%	Clopidogrel	24	34.3%	Azosemide	16	20.0%
Aspirin	81	15.0%	Azosemide	47	17.1%	Ferrous citrate	20	17.2%	Magnesium oxide	12	17.1%	Pantethine	14	17.5%
Bisoprolol	80	14.8%	Acetaminophen	37	13.5%	Lansoprazole	17	14.7%	Rosuvastatin	10	14.3%	Aspirin	13	16.3%
Azosemide	79	14.6%	Spironolactone	33	12.0%	Lebamipide	17	14.7%	Vonoprazan	10	14.3%	Ramelteon	13	16.3%
Clopidogrel	56	10.4%	Edoxaban	32	11.6%	Aspirin	14	12.1%	Sitagliptin	9	12.9%	Bisoprolol	12	15.0%
Edoxaban	54	10.0%	Aspirin	30	10.9%	Eldecalcitol	13	11.2%	Atorvastatin	7	10.0%	Spironolactone	11	13.8%
Lebamipide	53	9.8%	Isosorbide nitrate	29	10.5%	Alfacalcidol	11	9.5%	Azosemide	7	10.0%	Suvorexant	11	13.8%
						Metformin	11	9.5%	Bisoprolol	7	10.0%	Vonoprazan	11	13.8%
						Vonoprazan	11	9.5%	Cilostazol	7	10.0%			
									Edoxaban	7	10.0%			
									Pitavastatin	7	10.0%			

Table S3 Most common potentially inappropriate medications according to patient group

Total (n=541)			Subgroup											
			Subacute (n=275)			Post-acute orthopedics (n=116)			Post-acute neurological disorders (n=70)			Post-acute others (n=80)		
Azosemide	78	14.4%	Azosemide	47	17.1%	Celecoxib	22	19.0%	Aspirin	14	20.0%	Azosemide	16	20.0%
Spironolactone	52	9.6%	Spironolactone	33	12.0%	Metformin	11	9.5%	Clopidogrel	13	18.6%	Spironolactone	11	13.8%
Celecoxib	41	7.6%	Furosemide	22	8.0%	Azosemide	8	6.9%	Azosemide	7	10.0%	Aspirin	7	8.8%
Furosemide	32	5.9%	Celecoxib	16	5.8%	Famotidine	8	6.9%	Cilostazol	4	5.7%	Furosemide	7	8.8%
Metformin	30	5.5%	Metformin	15	5.5%	Eszopiclone	7	6.0%	Insulin sliding scale	4	5.7%	Clopidogrel	4	5.0%
Aspirin	25	4.6%	Risperidone	15	5.5%	Glimepiride	6	5.2%	Doxazosin	3	4.3%	Insulin sliding scale	3	3.8%
Clopidogrel	24	4.4%	Zolpidem	13	4.7%	Spironolactone	6	5.2%	Eszopiclone	3	4.3%	Magnesium oxide	3	3.8%
Famotidine	24	4.4%	Famotidine	11	4.0%	Zolpidem	5	4.3%	Famotidine	3	4.3%	Quetiapine	3	3.8%
Zolpidem	19	3.5%	Torasemide	11	4.0%	Clonazepam	4	3.4%	Metformin	3	4.3%	Loxoprofen	3	3.8%
Eszopiclone	18	3.3%	Magnesium oxide	10	3.6%	Etizolam	4	3.4%						
